# Inferring Species Trees from Gene Trees in a Radiation of California Trapdoor Spiders (Araneae, Antrodiaetidae, *Aliatypus*)

**DOI:** 10.1371/journal.pone.0025355

**Published:** 2011-09-26

**Authors:** Jordan D. Satler, James Starrett, Cheryl Y. Hayashi, Marshal Hedin

**Affiliations:** 1 Department of Biology, San Diego State University, San Diego, California, United States of America; 2 Department of Biology, University of California Riverside, Riverside, California, United States of America; Montreal Botanical Garden, Canada

## Abstract

**Background:**

The California Floristic Province is a biodiversity hotspot, reflecting a complex geologic history, strong selective gradients, and a heterogeneous landscape. These factors have led to high endemic diversity across many lifeforms within this region, including the richest diversity of mygalomorph spiders (tarantulas, trapdoor spiders, and kin) in North America. The trapdoor spider genus *Aliatypus* encompasses twelve described species, eleven of which are endemic to California. Several *Aliatypus* species show disjunct distributional patterns in California (some are found on both sides of the vast Central Valley), and the genus as a whole occupies an impressive variety of habitats.

**Methodology/Principal Findings:**

We collected specimens from 89 populations representing all described species. DNA sequence data were collected from seven gene regions, including two newly developed for spider systematics. Bayesian inference (in individual gene tree and species tree approaches) recovered a general “3 clade” structure for the genus (*A. gulosus*, californicus group, erebus group), with three other phylogenetically isolated species differing slightly in position across different phylogenetic analyses. Because of extremely high intraspecific divergences in mitochondrial COI sequences, the relatively slowly evolving 28S rRNA gene was found to be more useful than mitochondrial data for identification of morphologically indistinguishable immatures. For multiple species spanning the Central Valley, explicit hypothesis testing suggests a lack of monophyly for regional populations (e.g., western Coast Range populations). Phylogenetic evidence clearly shows that syntopy is restricted to distant phylogenetic relatives, consistent with ecological niche conservatism.

**Conclusions/Significance:**

This study provides fundamental insight into a radiation of trapdoor spiders found in the biodiversity hotspot of California. Species relationships are clarified and undescribed lineages are discovered, with more geographic sampling likely to lead to additional species diversity. These dispersal-limited taxa provide novel insight into the biogeography and Earth history processes of California.

## Introduction

Tremendous endemic diversity has evolved in the California Floristic Province. This diversity reflects many factors, including a relatively ancient continental margin landscape shaped by complex geologic events, resulting in very high topographic complexity, with correspondingly strong environmental and climatic gradients [1], [2]. These factors have promoted species diversification in numerous groups, and phylogenetic studies of such groups further inform our knowledge of Earth history processes in California [3]–[9]. A diverse component of the Californian fauna are the spiders in the suborder Mygalomorphae, which include tarantulas, trapdoor spiders, and kin. In terms of familial, generic, and species diversity, the Californian mygalomorph fauna is one of the richest in the world. This fauna is represented by eleven genera from the families Antrodiaetidae, Ctenizidae, Mecicobothriidae, Nemesiidae, Cyrtaucheniidae, and Theraphosidae. The antrodiaetids and cyrtaucheniids comprise the great bulk of the species diversity, and both families include genera that have radiated extensively and almost exclusively in California. The cyrtaucheniid genus *Aptostichus*, for example, includes over 30 species endemic to California [6], [10].

**Figure 1:**
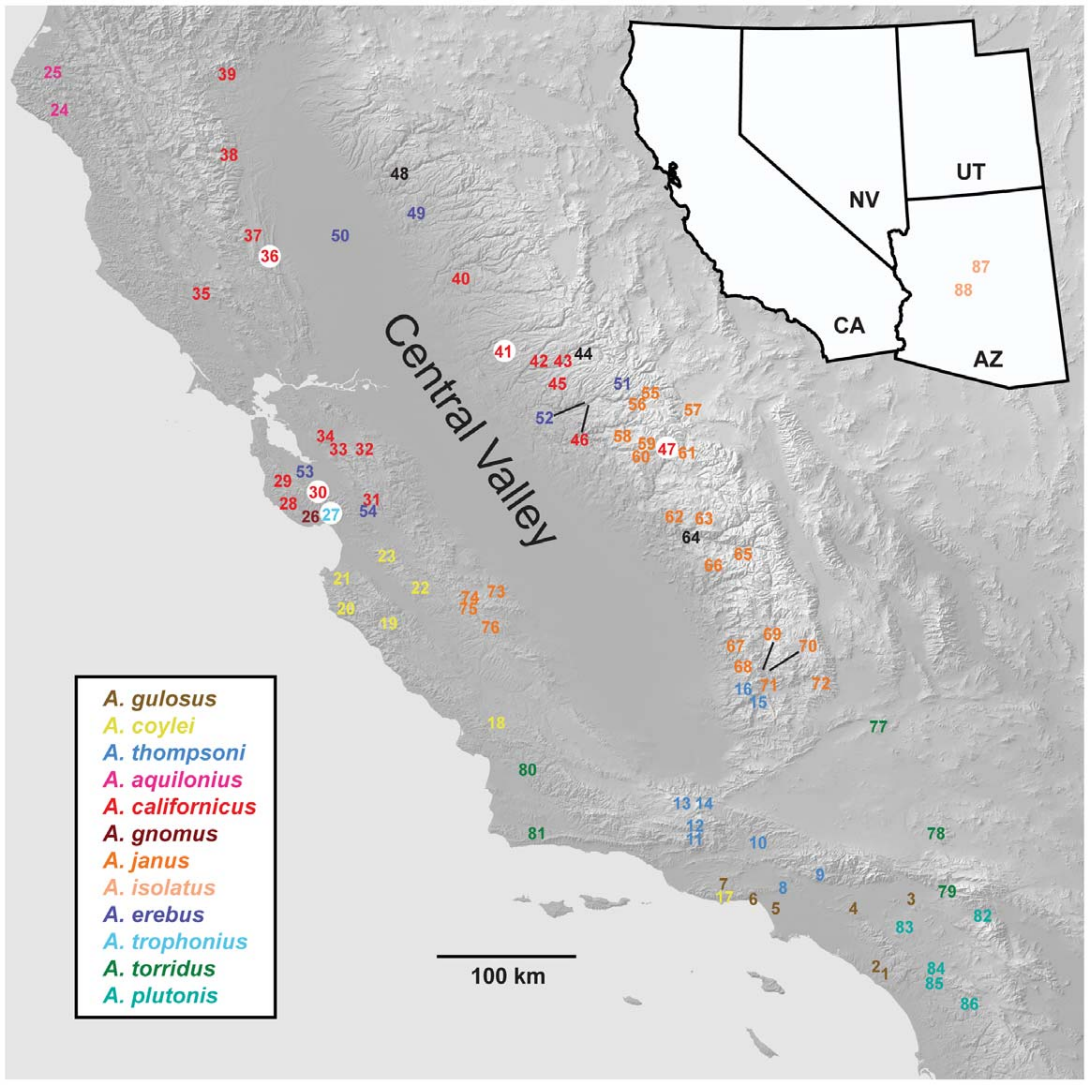
Map of California showing sampling localities, with colors corresponding to species (see insert). Sites with species syntopy highlighted with a white circle (27: *A. trophonius* & *A. californicus*; 30, 36, 41: *A. californicus* & *A. erebus*; 47: *A californicus* & erebus group immature). Black text designates locations where immature specimens could not be confidently identified. Detailed collection information can be found in Table S1.

The antrodiaetid genus *Aliatypus* is composed of twelve described species, eleven of which are endemic to California. *Aliatypus* are small- to medium-sized (∼6–20 mm) fossorial spiders that cover their subterranean burrows with a thin, flap-like trapdoor. Most Californian species occur in chaparral or mid-elevation forest habitats, although some species are also found in redwood forest or high-elevation pine forests, and a single species occurs southeast of the Sierra Nevada mountain range in high desert (Mojavean) habitats. The single non-Californian species is found in mid- to high-elevation forest of central montane Arizona. Species distributions (Fig. 1) range from relatively widespread taxa (e.g., *A. californicus*, *A. janus*, *A. erebus*), to more narrow endemic taxa (e.g., *A. gnomus*, *A. trophonius*, *A. aquilonius*). Most *Aliatypus* species occupy allopatric geographic distributions [11], [12], and all taxa show a general preference for cool, moist microhabitats (e.g., north-facing ravines, shaded roadcuts). This pattern of mostly exclusive geographic distributions and microhabitat specialization is seen in other well-studied California taxa, such as salamanders in the genus *Batrachoceps* [3], [13]. Diversification in Californian *Batrachoceps* has been described as a non-adaptive radiation dominated by vicariance and ecological niche conservatism, such that species are rarely found in syntopy (i.e., co-occurring at the same geographic location; [14]) despite considerable evolutionary age [3], [13].

Current hypotheses regarding species limits in *Aliatypus* are derived from the revisionary work of Coyle [11], who delineated species using a combination of morphological, geographical, and life history (i.e., burrow and trapdoor features) criteria. Coyle measured numerous morphological characters (both somatic and genital), and population variation was rigorously assessed to identify diagnostically informative characters. Coyle [11] noted considerable morphological geographic variation in some relatively wide-ranging species (e.g., *Aliatypus janus*, *A. thompsoni*). Also, the species *A. californicus* and *A. erebus* were hypothesized to include populations on both sides of the inhospitable Central Valley, an obvious modern-day and historical barrier to gene exchange (Fig. 1). Using a combination of phenotypic and natural history data, Coyle [15] placed *Aliatypus* species into three species groups, including the earliest diverging gulosus group (*A. gulosus*), the erebus group (*A. erebus, A. trophonius, A. torridus, A. plutonis*), and the californicus group (*A. californicus, A. gnomus, A. janus, A. isolatus, A. aquilonius*). The phylogenetic placement of *A. thompsoni* was essentially unresolved in these analyses. In addition, the newly described species *A. coylei* has yet to be phylogenetically placed, although Hedin & Carlson [16] suggest that this species is allied with the erebus group.

The resolution of species limits and interrelationships of mygalomorphs can be particularly challenging. Mygalomorph lineages tend to be morphologically conserved at shallow phylogenetic levels, leading to the potential underestimation of species diversity if taxonomy is based only on morphology [5], [6], [16]. At the same time, due to microhabitat specialization and limited dispersal abilities, these spiders tend to exhibit extreme population genetic fragmentation [5], [6], [17]. As such, single locus molecular studies (i.e., mitochondrial DNA only) are expected to potentially over-split diversity. Past studies have addressed these conflicting issues by using multiple lines of evidence, including multiple genes, morphology, and/or measurements of ecological niche divergence. As genomic resources become increasingly available for non-model taxa, multigenic phylogenetics becomes an obvious avenue for inferring species limits and interrelationships in mygalomorphs. Also, with the development of new analytical methods, systematists are transitioning from estimating gene trees to estimating species trees, and some are testing lineage hypotheses using explicit statistical approaches (summarized in [18]).

Here we use molecular data from one mitochondrial and six nuclear gene regions to address systematic and character evolution questions in *Aliatypus*. These data are used to address several problems involving species limits – for example, do geographically variable species include possible cryptic species? Can gene tree data be used to place morphologically “unidentifiable” immature specimens, and thus increase our knowledge of geographic distributions? Are short-range endemic species (*A. aquilonius, A. gnomus, A. trophonius*) genetically distinct from geographically neighboring wide-ranging species? What is the biogeographic influence of the Central Valley in species that apparently span this barrier? Finally, DNA sequence data are used (in concatenation and coalescent analyses) to infer an *Aliatypus* species tree, which is compared with prior morphological hypotheses, and is used to address the evolution of species syntopy in the genus.

## Materials and Methods

### Taxon Sample

*Aliatypus* specimens were collected from 89 geographic locations, with more sites sampled for taxa with larger geographic distributions (Fig. 1). Permits were attained for all pertinent California State Parks and National Parks. Our taxon sample provides comprehensive geographic coverage of the known range of all described species, including the recently described *A. coylei* [16], and includes noteworthy range extensions for several species (see Table S1). Examples include new western records of *A. janus* (sites 73–76, Fig. 1) and *A. torridus* (sites 80, 81; Fig. 1), and populations of *A. californicus* found much further north than previously documented (sites 38, 39; Fig. 1). At each location we attempted to collect adult spiders (almost always females), but sometimes collected only immature spiders, or collected a mixture of adults and immatures. Adult specimens were identified to species using somatic and genital morphology following Coyle [11] and Hedin & Carlson [16]; digital images of female spermathecal organs for all adult specimens have been deposited at Morphbank (www.morphbank.net).

A small number of immature spiders were identified based on geographic origin and/or association with adults from the same collection site. Most immatures were provisionally identified to species based on genetic information. To genetically identify (barcode) immature specimens we used the species delimitation plugin [19] for Geneious Pro v5.4.6 [20]. Specifically, we used this module to calculate the probability of a correct species identification (P ID (Liberal)) for unknowns (immature specimens), given a reference sequence alignment that includes identified (adult) specimens. These probabilities are based on the ratio of within-species genetic differentiation to the distance to the nearest species (*Intra/Inter* ratio). Ross et al. [21] found that this ratio predicted identification success as well or better than other metrics (e.g., “barcode gap”, BLAST, etc.), and these authors have derived number-of-taxa specific P ID (Liberal) values based on regression analyses of simulated data. For reference alignments we used the 28S and COI aligned matrices also used in individual gene phylogenetic analyses (see below).

Outgroup taxa were sampled following the phylogenetic hypotheses of Coyle [11] and Hendrixson & Bond [22]. The family Antrodiaetidae has traditionally included three extant genera, with *Aliatypus* placed sister to *Atypoides* and *Antrodiaetus*. Based on *Atypoides* paraphyly, Hendrixson & Bond [22] synonymized all *Atypoides* species with *Antrodiaetus*. To root our *Aliatypus* trees, we used sequences of both early diverging ( = *Atypoides*) and derived species of *Antrodiaetus* (see Table S1).

### Molecular Data Collection

Most spiders were transported live back to the lab, where entire legs were removed from freshly sacrificed specimens and preserved in 100% EtOH at −80°C. Voucher specimens were stored separately in 80% EtOH at −20°C. Genomic DNA was extracted from leg tissue using a DNeasy kit (Qiagen). Seven separate gene fragments were amplified via PCR (COI mtDNA, 28S rRNA, 18S rRNA, mitochondrial localized Hsp70 nDNA, EF-1γ nDNA, Fox-D nDNA, Wingless nDNA). Two of these gene regions (Hsp70, Fox-D) were newly developed from genomic resources for this study. Information regarding gene development, primers, and PCR protocols can be found in Table S2. PCR amplicons were directly sequenced in both directions, and contigs were assembled and edited using Sequencher 4.5 (Gene Codes Corporation, MI). For all nuclear genes except Hsp70, alleles were left unphased with heterozygous sites coded using standard ambiguity codes. For the Hsp70 data, PCR products containing length polymorphism were cloned and heterozygous individuals were sequenced for both alleles. We tested for recombination using TOPALi v2.5 [23], [24], implementing the DSS (Difference of Sums of Squares) method with default program settings.

### Sequence Alignment

To accommodate length variation found in the rRNA data (28S and 18S), we used the program MAFFT [25] using the G_INS-i alignment algorithm. MAFFT has been demonstrated to be effective at alignment of non-trivial rRNA gene sequences, with the G_INS-i algorithm being optimal for such sequences [26]. For the 28S MAFFT alignment we also used the program Gblocks [27] to remove regions of alignment uncertainty. This alignment was reduced from 1052 characters to 689 characters using a “less stringent” criterion (minimum number of sequences for a conserved position and flanking regions: 50; maximum number of contiguous non-conserved positions: 8; minimum length of a block: 5; allowed gap positions: with half).

### Phylogenetic Analyses

Models of DNA sequence evolution were selected with the program jModeltest 0.1.1 [28], [29] using the AIC criterion (models summarized in Table S2). Bayesian gene tree analyses were conducted on individual matrices using MrBayes v3.1.2 [30], [31]. The Bayes block consisted of the following parameters set as unlinked [revmat = (all), shape = (all), pinvar = (all), statefreq = (all), tratio = (all)]. For analyses containing multiple partitions, partitions were set as unlinked (ratepr = variable; see Table S2). Analyses were run for 2–6×10^6^ generations until reaching stationarity, initially assessed by an average standard deviation of split frequency under 0.01, then reviewed in Tracer v1.5 [32]. The first 40% of trees were discarded as burn-in, with remaining trees used to reconstruct a 50% majority rule consensus tree. Split frequencies were interpreted as posterior probabilities (pp) of clades.

It is well known that gene trees do not always reflect species relationships, due to errors in gene tree estimation, gene paralogy, introgression, and/or deep coalescence [18], [33], [34]. To estimate a species tree, we used both concatenation and coalescent-based approaches. In concatenation analyses samples were included when at least four loci were sampled for conspecific individuals from the same geographic location. In a small number of instances we concatenated genes from conspecific samples from different locations, where these samples were found to be phylogenetically close in single gene analyses (see Table S1). Although some taxa contained less than seven loci in the final concatenated matrix, this approach is relatively robust to missing data [35]–[37]. Data were partitioned by gene region (mtDNA COI additionally partitioned by codon position), with models assigned to partitions based on results from jModelTest, and analyzed using Bayesian search strategies as described above.

Estimating a species tree using concatenation has been shown to be potentially misleading under certain divergence scenarios [38], [39]. For example, Kubatko & Degnan [40] discuss multiple scenarios where concatenation may fail, including cases of recent or rapid speciation events, when internode branch lengths are short and deep coalescence is probable. In addition, concatenation constrains all loci to fit a single topology, an assumption clearly violated if independent gene regions have different evolutionary histories. As such, we also estimated a species tree using the program *BEAST [41], implemented in the BEAST v1.6.1 program package [42]. *BEAST operates under a Bayesian framework, jointly estimating the posterior distribution of species trees from the posterior distribution of individual gene trees using a coalescent model. *BEAST allows for gene tree heterogeneity, attributing gene tree/species tree discordance to deep coalescence. Missing data in coalescent-based species tree approaches may have a negative impact [34], [43]; if genealogies are missing for a taxon for a given gene, then a significant amount of information is missing when determining species relationships. To account for this, we limited *BEAST analyses to five genes (we lacked authentic 28S and 18S data for *Aliatypus aquilonius*, see Results). Following results from preliminary single gene analyses run in BEAST, we set the COI mtDNA marker (partitioned by codon position) to a relaxed uncorrelated lognormal clock; the remaining nuclear gene regions were set to a strict molecular clock. The molecular clock rate for EF-1γ was set to 1.0, with clock rates for remaining markers estimated under a gamma distribution. Models of DNA sequence evolution were assigned to each partition based on results from jModelTest. A Yule process was used for the species tree prior; the population size model was set to Piecewise linear and constant root. Default values were used for remaining priors. We ran *BEAST for 2×10^8^ generations with a sample frequency of 2×10^4^ generations. Convergence was assessed in Tracer v1.5, with the species tree reconstructed after a 40% burn-in using Tree Annotator v1.6.1 [42].

### Hypothesis Testing

To test particular phylogenetic hypotheses in the context of the well-sampled 28S and COI gene trees, we searched for specific taxon bipartitions in the 95% credible set of Bayesian trees [44], [45]. We first trimmed the unconstrained MrBayes trprobs file to include only the 95% credible set, then filtered this tree block in PAUP* 4.0 [46], applying filters conforming to the taxon bipartitions of Table 1.

**Table 1:** Results of Bayesian credible set hypothesis testing.

Hypothesis	COI Clade in 95% Credible Set (53,413 trees)	28S Clade in 95% Credible Set (79,630 trees)	ConcatenationClade in 95% Credible Set (42 trees)
**Monophyly** western *A. janus*	**NO - 0 trees**	**YES – 79488 trees**	**Not tested – too few samples**
**Monophyly** western *A. californicus*	**NO - 0 trees**	**NO - 0 trees**	**Not tested – too few samples**
**Monophyly** western *A. erebus*	**NO - 0 trees**	**NO - 0 trees**	**Not tested – too few samples**
**Monophyly** *A. janus*	**NO - 0 trees**	**YES – 48838 trees**	**NO - 0 trees**
**Monophyly** *A. californicus*+*A. gnomus*	**YES - 853 trees**	**YES – 79278 trees**	**YES – 42 trees**

## Results

### Data Availability

All specimens used in this study have been assigned a unique specimen identification number (MY or GMY; see Table S1). Upon completion of our on-going studies, a representative set of these voucher specimens will be deposited at the California Academy of Sciences, San Francisco, California. GenBank accession numbers for newly generated sequences are provided in Table S1. All seven gene regions contributed phylogenetic signal, i.e., parsimony informative sites, as follows: COI (402), 28S (282), 18S (235), Hsp70 (127), EF-1γ (83), Fox-D (40), and Wingless (25). TOPALi results suggest evidence of recombination in the 18S data (Table S3), although this is likely an artifact of high among-site rate variation [47]. Increasing window size and decreasing step size (as suggested by [48]) removed evidence for recombination in 18S. A Google Earth kmz file of all sampled locations is available upon request from the corresponding author.

### Individual Gene Tree Analyses

**Figure 2:**
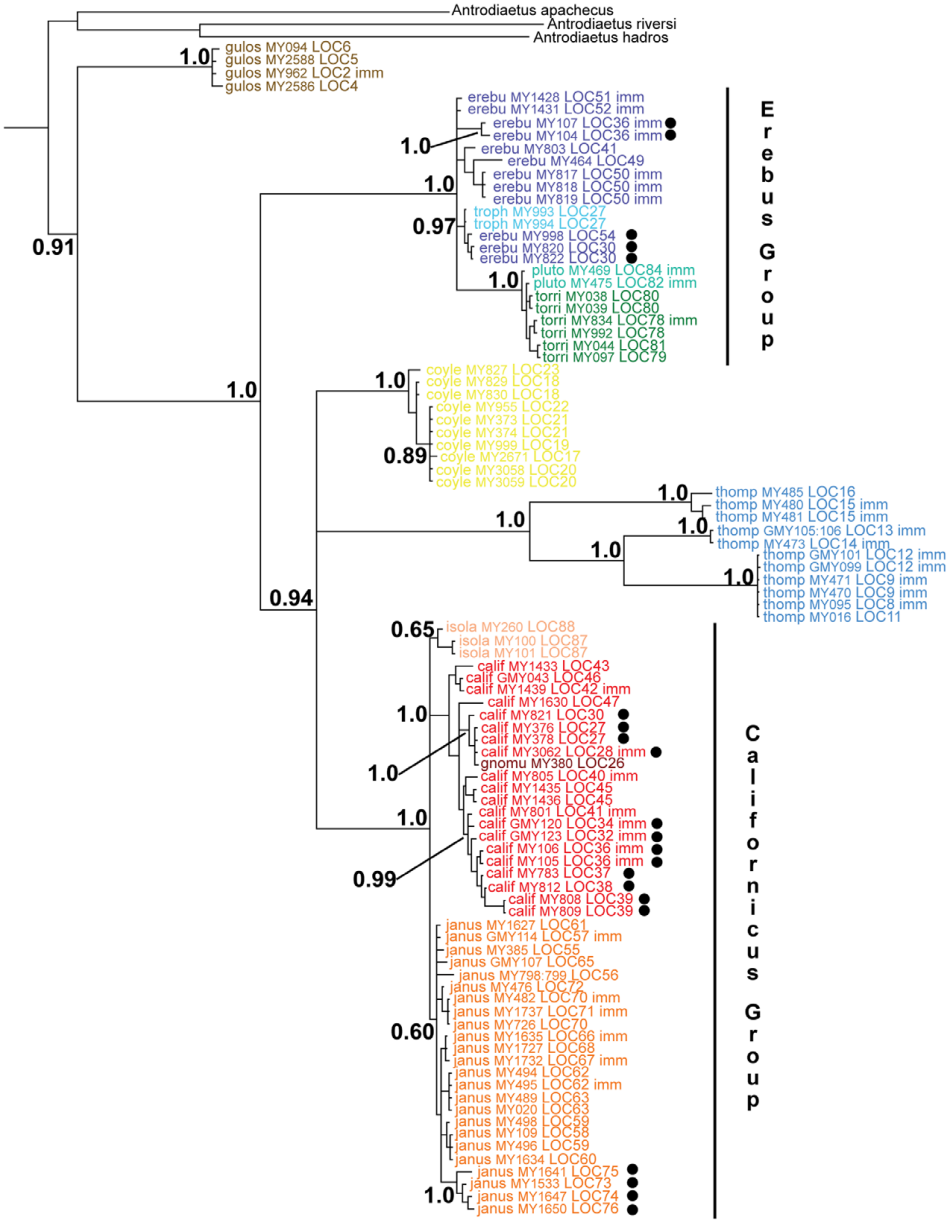
28S Bayesian phylogram. Posterior probabilities shown for all major clades. Taxon names consist of species, MY (or GMY) number, and collecting locality (see Fig. 1). Immature specimens are denoted with imm; western localities for transvalley taxa denoted with black dots.

The final aligned 28S matrix included ninety-five sequences collected from specimens representing 68 localities. The 28S and 18S sequences generated for multiple geographic samples of *A. aquilonius* included many unique singleton mutations. Alignment of these hypervariable sequences to other *Aliatypus* sequences was difficult, and this uncertainty in alignment prompted their removal from all analyses. A combination of long branches leading to different species, with relatively limited intraspecific divergence, results in significant (P>0.95) identification probabilities for most immature specimens with the 28S data (Table 2). This allowed immature species identification for one *A. gulosus*, one *A. torridus*, seven *A. erebus*, eight *A. thompsoni*, six *A. janus*, and eight *A. californicus* specimens. These genetic identifications are further supported by gene tree placement (Fig. 2), with most immatures falling into species clades anchored by adult (identified) specimens. These genetic identifications also make geographic sense, with immature specimens phylogenetically allied with samples from the same collection site or geographically adjacent sites. Although we lacked adult specimens, two immature specimens were *tentatively* identified as *A. plutonis* based on geographic distribution – these specimens were collected in a geographic region where *A. plutonis* is the only known *Aliatypus* taxon [11], [12].

**Table 2:** Species identification probabilities for immature specimens.

Species	Closest Species (Genetic Distance)	Monophyletic?	Intra/Inter	P ID (Liberal)
**28S Results**				
*A. gulosus*	*A. coylei*	yes	0.02	0.98 (0.87, 1.0)
*A. thompsoni*	*A. coylei*	yes	0.42	0.93 (0.87, 0.98)
*A. californicus* (*including *A. gnomus*)	*A. isolatus*	yes	0.53	0.96 (0.93, 0.99)
*A. janus*	*A. isolatus*	yes	0.58	0.95 (0.93, 0.98)
*A. torridus*	*A. erebus*	no	0.14	0.96 (0.86, 1.0)
*A. erebus* (*including *A. trophonius*)	*A. torridus*	no	0.35	0.96 (0.91, 1.00)
**COI Results**				
*A. thompsoni*	*A. janus*	yes	0.62	0.76 (0.65, 0.87)
*A. gulosus*	*A. thompsoni*	yes	0.34	0.92 (0.82, 1.0)
*A. aquilonius*	*A. janus*	yes	0.09	0.92 (0.77, 1.0)
*A. plutonis*	*A. torridus*	yes	0.63	0.65 (0.50, 0.80)
*A. torridus*	*A. plutonis*	no	0.62	0.76 (0.65, 0.87)
*A. erebus* (*including *A. trophonius*)	*A. torridus*	yes	0.64	0.89 (0.84, 0.95)
*A. californicus* (*including *A. gnomus*)	*A. janus*	no	0.79	0.89 (0.85, 0.94)
*A. janus*	*A. californicus*	no	0.85	0.87 (0.83, 0.91)

*Aliatypus gulosus* is recovered as sister to other species in the genus, with both the erebus and californicus groups recovered with strong support (Fig. 2). The californicus group is part of a larger clade including *A. thompsoni* and *A. coylei*, but the interrelationships of these three lineages are uncertain. Syntopy is restricted to taxa that are distantly related on the 28S tree (*A. erebus* & *A. californicus*: sites 30, 36, 41; *A. trophonius* & *A. californicus*: site 27).

The 28S data are variable and informative at the intraspecific level in *Aliatypus*, even after the Gblocks exclusion of many sites (from 1052 bp to 689 bp). Western samples of *A. janus* (in the south Coast Ranges) form a clade, nested within a larger Sierran (eastern) *A. janus* clade. This paraphyly suggests an east to west biogeographic directionality. Western samples of *A. californicus* fall into two clades, one consisting of three south Bay Area samples plus neighboring *A. gnomus*, and the other clade consisting of two Bay Area localities plus samples from the north Coast Ranges (Figs. 1 & 2). These western genetic clades are not sister to each other, and there are no trees in the 28S Bayesian credible set that support a single origin of western *A. californicus* (Table 1). In *A. erebus*, two south Bay Area samples form a clade that includes the geographically adjacent *A. trophonious*; western samples from the north Coast Ranges form a separate clade. Again, there are no trees suggesting western *A. erebus* monophyly in the 95% credible tree set (Table 1). Samples of *A. thompsoni* fall into three genetically divergent, geographically cohesive clades, with intraspecific 28S branch lengths conspicuously longer than many among-species branch lengths (Fig. 2).

**Figure 3:**
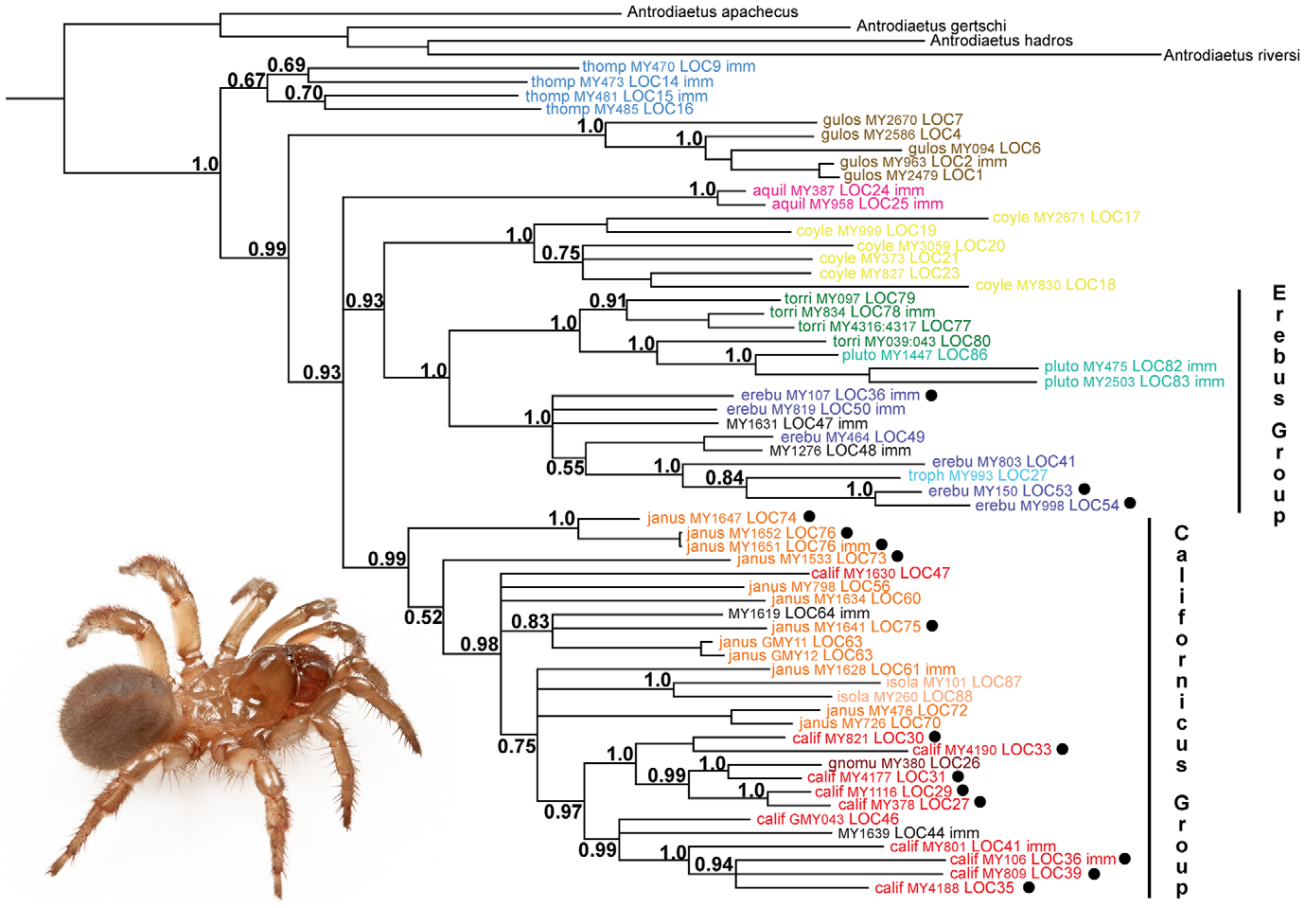
COI Bayesian phylogram. Posterior probabilities shown for all major clades. Taxon names consist of species, MY (or GMY) number, and collecting locality (see Fig. 1). Immature specimens are denoted with imm; western localities for transvalley taxa denoted with black dots. Taxon names in black are immatures that could not be confidently identified. Insert image of an adult female *Aliatypus thompsoni* from the central Transverse Ranges (Ventura Co., Cerro Noroeste Road).

Sixty-one COI mtDNA sequences were collected from specimens representing 55 localities; nineteen of these specimens were immature. Eight of these specimens were identified with high confidence using the 28S data, and their phylogenetic placement on the COI Bayesian gene tree is consistent with this identification (Fig. 3). We used geographic criteria to tentatively identify seven of the remaining eleven immature specimens – two specimens are identified as *A. aquilonius*, as these specimens are from known locations for this species, and this taxon is highly disjunct from all other *Aliatypus* species (Fig. 1). Three other immature specimens were identified based on association with identified specimens from the same geographic location (*A. gulosus* MY963, *A. janus* MY1651, *A. janus* MY1628), or exclusive geography (*A. plutonis* MY475, MY2503). All other immatures were conservatively deemed unidentifiable, reflecting low identification probability values (P<0.95) derived from the COI data (Table 2). These low values themselves reflect relatively high *Intra/Inter* ratios. As an example we highlight the californicus group, where average pairwise sequence divergence values (K2P model, [49]) within species in this group are very high (*A. janus* - 12.37%; *A. californicus* - 11.81%; *A. isolatus* - 11.28%; average within the californicus group - 13.03%; Table S4).

The COI tree recovers *Aliatypus thompsoni* as sister to other species in the genus (Fig. 3), contradicting the 28S hypothesis of *A. gulosus* as the basal member of the genus (Fig. 2). Both the californicus and erebus groups are recovered with strong support, with the latter group found sister to *A. coylei*; the position of *A. aquilonius* is unresolved. As is seen in the 28S data, syntopy is restricted to distant phylogenetic relatives (*A. erebus* & *A. californicus*: sites 36, 41, 47; *A. trophonius* & *A. californicus*: site 27).

As mentioned above, *Aliatypus janus* COI sequence clades are divergent and highly fragmented, and neither coastal nor Sierran localities are monophyletic. No trees were recovered supporting western *A. janus* monophyly in the 95% credible set (Table 1). For *A. californicus*, patterns in the COI gene tree mirror those seen in the 28S data, with localities sampled in the south Bay Area forming a clade that includes neighboring *A. gnomus*. A second clade of western *A. californicus* is recovered, and this clade is more closely related to eastern locales than to the other western clade. There are no trees with western *A. californicus* monophyly in the 95% credible set (Table 1). Coastal *A. erebus* samples from the south Bay Area form a clade with *A. trophonius*, independent of a single western locality from the north Coast Ranges. Credible set analysis again fails to recover trees supporting western *A. erebus* monophyly (Table 1).

**Figure 4:**
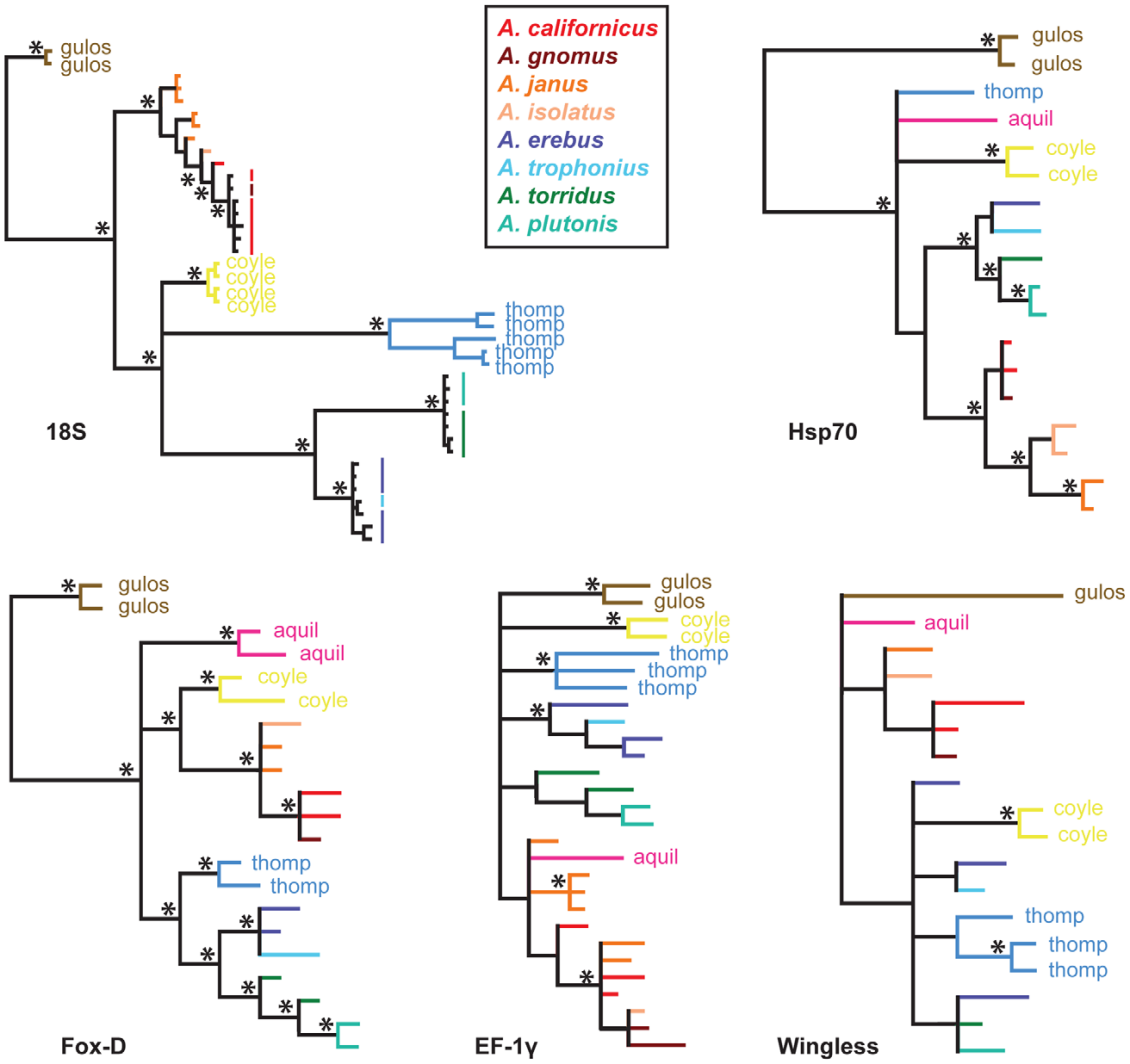
Bayesian phylograms from 18S rRNA, Hsp70 nDNA, Fox-D nDNA, EF-1γ nDNA, and Wingless nDNA matrices. Asterisks indicate pp values above 0.95%. The californicus and erebus group species are indicated by colored branches (see insert). Outgroups have been removed from gene trees for illustration purposes.

Results of Bayesian analyses for all remaining gene regions are shown in Fig. 4; in all cases these sequences were derived from adult or identifiable immature specimens. Sample sizes were smaller for these gene regions, with generally one to three sequences generated per species. Although these individual gene trees were sometimes characterized by weakly supported nodes, several congruent trends are apparent. *Aliatypus gulosus* is recovered as earliest diverging in three gene trees (Hsp70, Fox-D, 18S); all other gene trees recover a polytomy at the base of the genus. The erebus group is recovered in Hsp70, Fox-D, and 18S gene trees, while the californicus group is recovered in Hsp70, Fox-D, 18S, and Wingless gene trees. In general, the phylogenetic positions of *A. aquilonius*, *A. thompsoni*, and *A. coylei* vary among gene trees. For gene trees with sufficient geographic sampling, the phylogenetic positions of *A. gnomus* and *A. trophonious* are consistent with findings from both 28S and COI, which support *A. californicus* as paraphyletic with respect to *A. gnomus*, and *A. erebus* as paraphyletic with respect to *A. trophonius*.

### Species Tree Analyses

**Figure 5:**
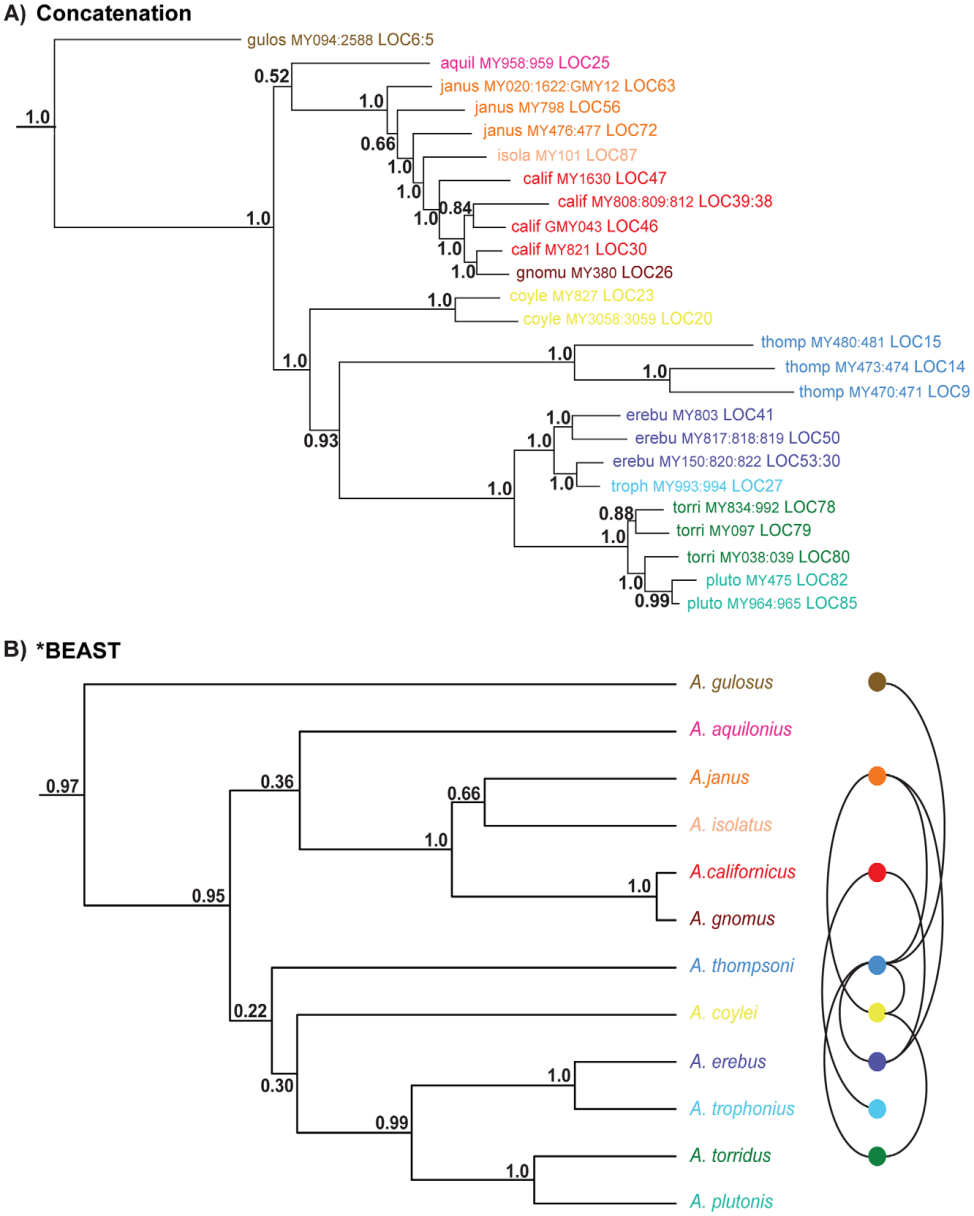
Species trees estimated with concatenation and coalescent approaches. Panel A: Bayesian phylogram of concatenated matrix; posterior probabilities shown for all nodes. Panel B: Phylogeny estimated with *BEAST; posterior probabilities shown for all nodes. Patterns of syntopy displayed for *Aliatypus*, corresponding to Table 3. Outgroups have been removed from species trees for illustration purposes.

The concatenated matrix includes data for 25 terminals; four terminals were sampled for all seven gene regions (5656 bp), with remaining terminals missing data for at least one gene region. Bayesian analysis of this matrix recovers *Aliatypus* monophyly with strong support (pp = 1.0), with *A. gulosus* placed as sister to the rest of the genus (Fig. 5A). The californicus group is recovered with strong support (pp = 1.0), although interrelationships within this group are less clear. *Aliatypus janus* is recovered as earliest diverging and paraphyletic, suggesting possible cryptic species diversity. *Aliatypus californicus* is paraphyletic with respect to *A. gnomus*, consistent with single gene analyses. The erebus group is well supported (pp = 1.0), with species recovered in two sister clades, including *A. erebus* plus *A. trophonius*, and *A. torridus* plus *A. plutonis*. *Aliatypus erebus* is paraphyletic with respect to *A. trophonius*, and *A. torridus* is paraphyletic with respect to *A. plutonis*. The phylogenetic positions of *A. thompsoni* and *A. coylei* are strongly supported, with *A. thompsoni* sister to the erebus group, and *A. coylei* sister to this larger clade. The placement of *A. aquilonius* as sister to the californicus group is weakly supported (pp = 0.52), consistent with the variable placement of this species in individual gene analyses.

The *BEAST analysis recovers a similar topology to the concatenated analysis, although some discordance is seen (Fig. 5B). Both the californicus and erebus groups are recovered as monophyletic with strong support. Within the californicus group, *A. janus* is recovered as sister to *A. isolatus*, contrasting with the results of concatenation. *Aliatypus coylei* is recovered as sister to the erebus group, with *A. thompsoni* sister to the *A. coylei*/erebus group clade, although these relationships are weakly supported (pp = 0.30 and 0.22). *Aliatypus aquilonius* is recovered as sister to the californicus group with low support (pp = 0.36). Patterns of species syntopy, based on this research and prior studies (Table 3), are summarized on the *BEAST tree (Fig. 5B). This summary clearly shows that syntopy in *Aliatypus* is restricted to distant phylogenetic relatives. For example, there is no known syntopy within the californicus and erebus species groups, despite many examples of species parapatry in these groups (Fig. 1).

**Table 3:** Patterns of syntopy in *Aliatypus*.

Species Combinations	References/Locations
*A. californicus* & *A. erebus*	sites 30, 36, 41 – this study; [12]
*A. californicus* & erebus group immature	site 47 – this study
*A. californicus* & *A. trophonius*	site 27 – this study; [12]
*A. janus* & *A. erebus*	[12]
*A. janus* & *A. thompsoni*	34.3532–120.2633 (per. obs.)
*A. thompsoni* & *A. gulosus*	34.1581–119.0033 (per. obs.)
*A. thompsoni* & *A. torridus*	34.6785–119.3626 (per. obs.)
*A. thompsoni* & *A. coylei*	34.8137–118.8904 (per. obs.)
*A. thompsoni* & *A. erebus*	35.5869–118.4383 (per. obs.)
*A. coylei* & *A. torridus*	34.8624–119.1275 (per. obs.)
*A. coylei* & *A. janus*	[16]

## Discussion

### Aliatypus Species Tree

One goal of this study was to estimate an *Aliatypus* species tree from multilocus data, and to compare this molecular phylogenetic perspective with previous morphology-based hypotheses [11], [15]. In general, these prior hypotheses are supported by molecular data, with some exceptions. Previous work suggests californicus group monophyly based on “female tibia IV relatively short,” and a sister relationship for *A. aquilonius* with *A. isolatus*/*A. janus* based on “spermathecal stalks tapered” [15]. However, *A. aquilonius* is not strongly supported as a member of the californicus group, suggesting homoplasy in at least the spermathecal characters. Given the simplicity of *Aliatypus* spermathecal organs (thin cuticular tubes with a terminal bulb; see [11]), this homoplasy is perhaps not surprising. Within the californicus group, concatenation suggests *A. janus* to be the earliest diverging member. *Aliatypus isolatus* is found to diverge next, sister to an *A. californicus*/*A. gnomus* clade. This suggests that the shared spermathecal characteristics of *A. isolatus* and *A. janus* perhaps represents a plesiomorphic condition, but we note that *BEAST analyses recover *A. isolatus* and *A. janus* as sister species, although with weak support (pp = 0.66). Lastly, as in previous phylogenetic studies [11], [15], this study fails to resolve the phylogenetic placement of *A. thompsoni*. A distant relationship with the erebus group is supported by concatenation, but this contrasts with an unresolved placement in *BEAST analyses. Relatively short internal branches may be causing the discordance between these two methods, as this divergence history (e.g., rapid speciation) has been shown to be problematic for species tree reconstruction [38], [50].

Accurate identification of morphologically unidentifiable immatures was vital to the study, since it was not always possible to collect adult spiders from all locations. We note that this rarity of adult specimens is a general issue in mygalomorph systematics – these reclusive spiders are simply often difficult to find in large numbers, and as adults. For this study, explicit COI-based genetic identification [19], [21], [51], [52], [53] was found to be rather uninformative (Table 2). Instead of a pattern of low intraspecific divergence and high interspecific divergence (i.e., a barcoding “gap”), we found intraspecific divergences approaching those observed among species (e.g., within *A. californicus* - 11.81%; within the californicus group - 13.03%; K2P model; see Table S4). This lack of a barcoding gap seems to be a general characteristic of mitochondrial sequence data in dispersal-limited mygalomorphs (e.g., [5]).

In contrast to other arthropod studies (e.g., [54]–[56]), we found the 28S gene region to be more informative than COI for immature specimen identification (Table 2). Relatively long branches separate 28S species groupings, allowing more confidence in species identification of specimens. This finding is not completely unexpected, as rate acceleration of 28S has been hypothesized for antrodiaetids [57], and prior species-level work in antrodiaetids has shown the utility of this gene region [5], [22]. This result was seen for *Aliatypus* even after the Gblocks removal of ambiguous alignment regions in 28S; at intraspecific levels where alignment may not be as problematic, the use of a greater percentage of data would be expected to provide even further resolution.

### TransValley Biogeography

A prominent geographic feature of the California landscape is the Central Valley (see Fig. 1), comprising a ring of mesic habitats (Sierra Nevadas, Transverse Ranges, Coast Ranges) surrounding relatively more xeric habitats. These landscape features have led to a “ring-like” distribution in many species, where upland taxa are currently absent or greatly limited in the Central Valley [8], [58]–[60]. However, while the modern Central Valley is clearly inhospitable for many species (minimal topographic relief combined with nearly complete land conversion), past conditions have apparently been sporadically suitable for existence in and/or dispersal across the valley [61], [62]. Evidence for this suitability is seen in multiple species of forest-dwelling upland salamanders, including *Batrachoseps* [3], [13], [63], *Ensatina* [8], [64], [65], and *Aneides* [66]. These genera include species with transvalley disjunct distributions in central California (at latitudes near San Francisco). Genetic studies of these species reveal a west-to-east “transvalley leak,” with low levels of genetic divergence in the Sierra Nevadas suggesting a recent eastward expansion during the Pleistocene.

Three *Aliatypus* species (*A. janus, A. californicus, A. erebus*) show disjunct transvalley distributions, providing potential replicated cases of transvalley dynamics. In *A. janus*, we discovered new western populations in the southern Coast Ranges. These samples form a clade in 28S analyses, and the gene tree topology suggests an east to west directionality. However, the deeply divergent COI data do not show this same pattern. As the COI data is expected to coalesce faster than 28S (smaller effective population sizes of mtDNA vs. nDNA), deep coalescence seems implausible, and mutational saturation of the COI data may be a better explanation for this discordance. Also, the 28S western clade may be an artifact of a sampling gap, but despite extensive sampling in the uplands south of the Central Valley between the south Coast Ranges and Sierra Nevadas, no *A. janus* specimens have been collected. This would suggest that the disjunct distribution is real, with a single crossing of the Central Valley the most likely scenario. The mechanism of crossing, i.e., long-distance dispersal versus contiguous range expansion with subsequent vicariance, remains to be determined.

We recovered multiple clades of western *A. erebus* and *A. californicus* in individual gene tree analyses of the 28S and COI data. These western groups do not form a single clade in either species, with their position in gene trees suggesting an east to west directionality with multiple “transvalley” events for both species. This finding is in contrast with the salamander studies, both temporally (based on levels of sequence divergence) and directionally. Patterns seen in both *A. californicus* and *A. erebus* support the central Sierra Nevada foothills as close to the area of dispersal for these species across the Central Valley. Despite the opposite directionality, the general latitudinal position of the “transvalley leak” seen in *A. californicus* and *A. erebus* is consistent with that seen in salamander studies. The east to west directionality observed in *Aliatypus* is also consistent with phylogeographic findings in *Antrodiaetus riversi* [5].

### Cryptic Species?

Multiple *Aliatypus* species occupy relatively large geographic distributions, increasing the likelihood of undiscovered diversity in the form of cryptic species. Cryptic species sometimes show slight morphological differences that only become “significant” when viewed in a *post hoc* manner (i.e., after DNA evidence suggests the boundaries of such lineages). We hypothesize that several cryptic *Aliatypus* species are represented in our current sample, and highlight two examples here, although others likely exist. Cryptic species are probable in *A. janus*, as suggested by the pattern of geographically distinct, early diverging lineages creating paraphyly in most individual gene tree analyses and the concatenated analysis. Another species that likely harbors undiscovered species diversity is *A. thompsoni*. Clearly a monophyletic group, high internal divergence (Table 2) and consistent recovery of three divergent clades in individual (e.g., Fig. 2) and concatenated analyses suggest independently evolving lineages. These lineages are also geographically cohesive and spatially isolated, distributed in the Transverse Ranges, Tehachapi Mountains, and southern Sierra Nevada Mountains, a well-known diversification hotspot [3], [7], [67], [68].

Because of limited dispersal abilities and morphological conservation, cryptic species are common in mygalomorphs. This finding certainly holds true in California, where basically all studies of California mygalomorphs have revealed cryptic lineages within morphologically defined species [5], [6], [16], [69], [70]. This discovery of new species of relatively large, “charismatic” spiders is noteworthy, as they are found in a biodiversity hotspot and a geographic region assumed to be “well-known.” We noted probable cases of cryptic speciation above, but believe that denser geographic sampling could lead to the discovery of additional short-range endemic species in *Aliatypus*. Overall, the California mygalomorph fauna represents a compelling framework for understanding the problem of species delimitation in dispersal-limited taxa, particularly in the context of new data generation technologies (e.g., next-generation sequencing) and new analytical methods [18], [41], [71], [72].

### Speciation in the Redwoods?

The currently recognized Bay Area species *A. gnomus* and *A. trophonius* are redwood forest short-range endemics (see Fig. 1; [11]). *Aliatypus gnomus*, most closely related to *A. californicus*, was originally described based on 8 adult specimens from a single location in the southern Santa Cruz Mountains. *Aliatypus trophonius*, most closely related to *A. erebus*, was described from two localities (14 adult specimens) in the same region [11]. Both of these taxa are hypothesized to have diverged via adaptive speciation, with the evolution of both small body size and short burrow depth resulting in an adaptive shift [15]. Because of surface to volume ratio relationships, this morphological and ecological divergence now constrains these taxa to relatively moist redwood forest habitats [15]. Both species are also distinct in genital morphology [11].

Based on molecular data, both species render neighboring populations of other species paraphyletic. For *A. trophonius*, we see the same pattern across four loci (COI, 28S, 18S, EF-1γ), with alleles related to Bay Area samples of *A. erebus*. *Aliatypus gnomus* shows a similar pattern, falling into a clade of Bay Area *A. californicus* for COI and 28S. This gene tree paraphyly is consistent with two alternative hypotheses. First, the molecular data may indicate that the diminutive redwood species are not actually unique species, but rather are geographic variants of more widespread taxa. We note here that neighboring *A. californicus* and *A. erebus* populations have also been collected in redwood forests, so it is not occurrence in redwood habitats *per se* that distinguishes the small-bodied species. However, as noted above, the small-bodied species are behaviorally distinct (building shorter burrows), and fall outside of the range of known morphological geographical variation for either *A. californicus* or *A. erebus* [11].

The alternative hypothesis involves an adaptive shift as envisioned by Coyle [11], but one recent enough such that not enough time has occurred for alleles of *A. californicus* and *A. erebus* to sort to monophyly. Unfortunately, our current sample of a single collection locality for each redwood endemic limits our ability to test these alternative hypotheses. As these species are hypothesized to have evolved via adaptive divergence, future studies should strive to collect sufficient genetic data to statistically test different divergence scenarios, which could provide insight into the mode and timing of speciation for these redwood endemics [9]. Additionally, these data could be used to test if these species diverged in isolation, or if they have diverged with gene flow [73], [74]. Such data and analyses might also allow us to tease apart the temporal distribution of gene flow (i.e., initial gene flow followed by isolation versus continuous gene flow throughout speciation).

Patterns seen in the redwood endemic *Aliatypus* species are paralleled in other antrodiaetid taxa. Hendrixson & Bond [75] demonstrated *Antrodiaetus unicolor* to be paraphyletic with respect to *An. microunicolor*. The latter species is morphologically distinct, exhibits extreme reduction in body size, and is a micro-endemic taxon geographically nested within the range of the more wide-ranging *An. unicolor*.

### The Evolution of Syntopy

For the purposes of this study, we define syntopy as the co-occurrence of species at a single collecting location [14], acknowledging that this single location may include multiple microhabitats. Coyle & Icenogle [12] argue that syntopy in *Aliatypus* is rare, and when evident, restricted to distantly related species (see fig. 1 of Coyle & Icenogle [12]). Our research confirms this pattern, showing that syntopy is restricted to distant relatives (see Fig. 5B; Table 3). In particular, we never find species from the same species group (e.g., californicus group, erebus group) together at the same geographic location.

We argue that this phylogenetically informed ecological pattern supports a model of allopatric speciation with niche conservation [76], [77]. Under this model, closely related species occupy essentially equivalent subterranean niche space, precluding syntopy [3], [13]. Niche divergence is conservative but accumulates with time, such that distantly related species can ultimately co-exist in syntopy. In *Aliatypus*, potential niche differentiation may result from divergence in burrow features (e.g., depth, diameter, etc.) and door shapes which themselves impact microhabitat preferences [12]. We argued above that the redwood species *A. gnomus* and *A. trophonius* may have evolved via ecological speciation. However, the fact that we never find these species in syntopy with close relatives (*A. californicus*, and *A. erebus*, respectively) is more consistent with the niche conservation speciation model.

Two arguments against the niche conservation model must be considered. The first relates to time since divergence - perhaps not enough time has passed for close relatives to move into sympatry, precluding potential observations of syntopy. The second confounding factor is that closely related species of *Aliatypus* (i.e., from the same species group) may not have evolved complete reproductive isolation. If hybridization or introgression or a combination of the two has occurred, we may not be able to readily observe syntopy between closely related species. This is because hybrids may be difficult to identify with morphology, and molecular data may be inconclusive as to species placement.

Geographic patterns observed in *Aliatypus* are similar to those found in Californian *Batrachoseps* salamanders, where species have diverged via allopatric speciation, with niche conservatism apparently preventing syntopy between genetically divergent lineages. Because different *Batrachoseps* species are ecologically very similar, species are more likely to replace one another geographically rather than partition niche space and co-occur in the same habitat. Thus, most geographic locations include only a single *Batrachoseps* species, with already established taxa resisting invasion from other species (termed “preemptive occupancy of space,” [13]).

### Conclusions

This work builds upon previous studies of this diverse group of trapdoor spiders [11], [12], [15], [16], and more generally, provides insight into divergence dynamics within the Californian arthropod fauna. Species relationships within the genus are further clarified, including the placement of a newly described species (*A. coylei*). The complex landscape that characterizes California creates numerous opportunities for population isolation in dispersal-limited taxa. This is clear in *Aliatypus*, as evidenced by the high number of described species and potential cryptic species, and also in patterns of deep population-level genetic divergence. We argue that dispersal-limited taxa offer an advantage in studies of biogeography, as their sedentary lifestyle makes them more likely to reveal Earth history processes. This study shows the utility of *Aliatypus* in such studies, providing new insights into California biogeography. We hope that this work stimulates further investigation into this diverse group of trapdoor spiders.

## Supporting Information

Table S1 **Specimen information and GenBank accession numbers.**
(XLS)

Table S2 **Molecular data information.**
(XLS)

Table S3 **Results of recombination testing.**
(XLS)

Table S4 **Average K2P corrected sequence divergence.**
(XLS)
